# A Probiotic Intervention With Bifidobacterium longum NCC3001 on Perinatal Mood Outcomes (PROMOTE Study): Protocol for a Decentralized Randomized Controlled Trial

**DOI:** 10.2196/41751

**Published:** 2023-04-05

**Authors:** Melissa P S Toh, Chui Yuen Yang, Phei Cze Lim, Hui Li J Loh, Gabriela Bergonzelli, Luca Lavalle, Elias Mardhy, Tinu Mary Samuel, Elvira Suniega-Tolentino, Irma Silva Zolezzi, Lisa R Fries, Shiao Yng Chan

**Affiliations:** 1 Singapore Institute for Clinical Sciences Agency for Science, Technology and Research Singapore Singapore; 2 Nestle Research, Lausanne Lausanne Switzerland; 3 Nestle Research, Singapore Singapore Singapore; 4 Nestle Research, Beijing Beijing China; 5 Department of Obstetrics and Gynaecology Yong Loo Lin School of Medicine National University of Singapore Singapore Singapore

**Keywords:** perinatal mood disturbances, pregnancy, randomized clinical trial, low mood, stress, anxiety, depression, probiotics, mobile phone

## Abstract

**Background:**

Perinatal mood disorders such as depression and anxiety are common, with subclinical symptomology manifesting as perinatal mood disturbances being even more prevalent. These could potentially affect breastfeeding practices and infant development. Pregnant and lactating women usually limit their exposure to medications, including those for psychological symptoms. Interestingly, the naturally occurring probiotic *Bifidobacterium longum* (BL) NCC3001 has been shown to reduce anxious behavior in preclinical models and feelings of low mood in nonpregnant human adults. During the COVID-19 pandemic, mental health issues increased, and conventionally conducted clinical trials were restricted by social distancing regulations.

**Objective:**

This study, Probiotics on Mothers’ Mood and Stress (PROMOTE), aimed to use a decentralized clinical trial design to test whether BL NCC3001 can reduce symptoms of depression, anxiety, and stress over the perinatal period.

**Methods:**

This double-blind, placebo-controlled, randomized, and 3-parallel-arm study aimed to recruit 180 women to evaluate the efficacy of the probiotic taken either during pregnancy and post partum (from 28-32 weeks’ gestation until 12 weeks after delivery; n=60, 33.3%) or post partum only (from birth until 12 weeks after delivery; n=60, 33.3%) in comparison with a placebo control group (n=60, 33.3%). Participants consumed the probiotic or matched placebo in a drink once daily. Mood outcomes were measured using the State-Trait Anxiety Inventory and Edinburgh Postnatal Depression Scale questionnaires, captured electronically at baseline (28-32 weeks’ gestation) and during e-study sessions over 5 further time points (36 weeks’ gestation; 9 days post partum; and 4, 8, and 12 weeks post partum). Saliva and stool samples were collected longitudinally at home to provide mechanistic insights.

**Results:**

In total, 520 women registered their interest on our website, of whom 184 (35.4%) were eligible and randomized. Of these 184 participants, 5 (2.7%) withdrew after randomization, leaving 179 (97.3%) who completed the study. Recruitment occurred between November 7, 2020, and August 20, 2021. Advertising on social media brought in 46.9% (244/520) of the prospective participants, followed by parenting-specific websites (116/520, 22.3%). Nationwide recruitment was achieved. Data processing is ongoing, and there are no outcomes to report yet.

**Conclusions:**

Multiple converging factors contributed to speedy recruitment and retention of participants despite COVID-19–related restrictions. This decentralized trial design sets a precedent for similar studies, in addition to potentially providing novel evidence on the impact of BL NCC3001 on symptoms of perinatal mood disturbances. This study was ideal for remote conduct: because of the high digital literacy and public trust in digital security in Singapore, the intervention could be self-administered without regular clinical monitoring, and the eligibility criteria and outcomes were measured using electronic questionnaires and self-collected biological samples. This design was particularly suited for a group considered vulnerable—pregnant women—during the challenging times of COVID-19–related social restrictions.

**Trial Registration:**

ClinicalTrials.gov NCT04685252; https://clinicaltrials.gov/ct2/show/NCT04685252

**International Registered Report Identifier (IRRID):**

DERR1-10.2196/41751

## Introduction

### Background

Anxiety and depression during pregnancy and post partum are referred to as perinatal mood disorders [1-3]. Subclinical symptomatology, including low mood and stress, that does not reach thresholds for clinically diagnosable pre- and postpartum depression and anxiety is also becoming an area of concern. Collectively, such symptoms of varying severity in the perinatal period will be referred to as perinatal mood disturbances (PMD) in this paper. Depressive symptoms are relatively common in the perinatal period, and many women experience mild forms of such negative feelings, typically called *baby blues*, owing to many factors, including physiological hormonal changes [1-5]. It is estimated that as many as 70% of women experience “baby blues” during the postpartum period [4], and approximately 24% of women in their third trimester of pregnancy and 15% of postpartum women experience anxiety [6-8]. Furthermore, low mood and stress often coexist in the postpartum period [4]. The consequences of PMD, even within normal variation of mood and stress, can also have a downstream impact on the infant’s development and behavior owing to alterations in the in utero environment [1-3,8] and in postnatal mother-infant interactions [9,10]. In addition, postpartum mood disorders are reported to be negatively associated with the initiation and duration of breastfeeding [1-4,10,11], which are known to have multiple short- and long-term consequences on maternal and infant health [11,12].

Many women opt to avoid taking pharmacological treatments to address feelings of low mood and stress during the perinatal period [1,13], often to prevent the potential risk of drugs being transferred to the baby through the placenta or breast milk. Thus, there is a specific need for alternative solutions for pregnant and breastfeeding mothers [14]. Currently, many pregnant women are open to consuming naturally occurring probiotics, which are emerging as a potential solution to modulate mood. The concept of a gut-brain axis linking differences in gut microbiome with central nervous system function via endocrine factors, inflammatory mediators, neurotransmitters, and autonomic nervous signaling is becoming more compelling and could explain how probiotics may affect emotions and mood [15].

A randomized controlled trial of the probiotic *Lactobacillus rhamnosus* HN001, whose primary outcome was infant eczema, showed its potential role in reducing PMD as a secondary outcome [16]. However, the strength of the conclusions was limited by the lack of baseline mood scores, mood evaluation at a single time point, and the retrospective collection of mood data 4 to 11 months post partum potentially contributing to recall bias. A more recent study, which assessed the effect of a multiple probiotic strain combination of *Lactobacillus rhamnosus* GG and *Bifidobacterium lactis* BB12 administered to pregnant women with obesity between 12 and 36 weeks of pregnancy, showed no difference between the intervention and placebo groups in improving prenatal depression or anxiety [17]. Other probiotic trials in pregnancy also found no statistically significant effects on PMD [18,19]. Such mixed findings suggest variation in the impact of different probiotics on PMD, consistent with findings of strain-specific effects of probiotics on other health outcomes [20].

Outside of the perinatal setting, the naturally occurring probiotic *Bifidobacterium longum* (BL) NCC3001 normalized anxiety-like behavior in different preclinical models [21,22] and reduced feelings of low mood and emotional reactions to fearful stimuli in human adults with irritable bowel syndrome [23]. However, a possible effect of this probiotic during the perinatal period has not yet been tested.

This study was designed to test the efficacy of the probiotic BL NCC3001 as a nonpharmacological intervention in reducing PMD. In addition, we are investigating the optimal time window for commencement of probiotic intake to obtain a protective effect: whether there is a need to start the intervention prenatally or whether there would still be a beneficial effect if it is started only after delivery. We hypothesize that daily ingestion of the probiotic BL NCC3001 in the perinatal period will lower time-trend scores of mood and stress as measured by the validated State-Trait Anxiety Inventory (STAI) [24] and Edinburgh Postnatal Depression Scale (EPDS) [25] questionnaires.

### Decentralized Study

With the unprecedented advent of the COVID-19 pandemic at the start of 2020, the conduct of many clinical trials was hampered by government-imposed restrictions on face-to-face contact. It was particularly challenging to set up new trials to evaluate interventions unrelated to COVID-19 infections, especially in populations considered vulnerable, such as pregnant women. Clinical trials that could be designed for remote conduct using digital tools were at an advantage [26]. Given that our intervention could be self-administered without clinical monitoring and that the primary outcome was measurable using electronic questionnaires, the design allowed for decentralized remote conduct. This approach was thought to be also well suited to the advanced, high-resource setting of Singapore where participants have easy access to the technology needed for remote contact with study teams. Here, we present the protocol of an entirely decentralized study in Singapore, the Probiotics on Mothers’ Mood and Stress (PROMOTE) study, which could serve as a basis for the design of similar studies in the future.

## Methods

### Design

Study aspects as well as the technical means used for each aspect are presented in Textbox 1.

**Study aspect and technical means used for each aspect of this decentralized study.**

**Recruitment**

- Digital advertising (social media, parenting websites, and workplace website)
- Physical posters in public places (public transport and stores)
- Paper brochure dissemination in mailboxes and partner locations
- Word of mouth

**Registration of interest**

- Secured online form; participant-initiated self-referral

**Screening and study sessions**

- Video call with screen-sharing capabilities

**Signing of the informed consent form**

- REDCap (Research Electronic Data Capture; Vanderbilt University) electronic database

**Delivery of intervention product and study materials, as well as biological sample collection**

- Trained and licensed courier service

**Self-reported questionnaires**

- Medidata Patient Cloud (Medidata Solutions Inc) mobile app in which participants can complete questionnaires (automatically syncs to iMedidata; Medidata Solutions Inc)

**Product compliance and all non–self-reported data**

- iMedidata electronic patient-reported outcome database entry completed by study team

### Recruitment and Remote Study Conduct

This trial was set up and conducted in Singapore, where the smartphone penetration rate in the population is 98% and where 42.5% of the population, including 34.3% of women of reproductive age, use social media predominantly to research brands [27]. There is also a high level of trust in digital security systems associated with governmental organizations such as the Agency for Science, Technology and Research (A*STAR).

Recruitment in the community at large in Singapore was carried out via self-referral by interested women who heard about the study through the following channels: (1) social media advertising; (2) parenting websites as well as internet forums, platforms, and group chats; (3) physical posters on public transport; (4) brochures in mailboxes and partner locations; and (5) word of mouth. Relevant groups were approached through strategies such as targeted advertising to female users of reproductive age and specific estimated due date groups on various social media and messaging platforms, sending out electronic direct mail messages to members of the parenting websites engaged for the study, and placing physical posters in maternity stores or electronic posters on relevant websites such as the A*STAR Singapore Institute for Clinical Sciences web page.

All advertisements included the distinctive, easily recognizable PROMOTE logo. Electronic advertisements contained a convenient web link, and public posters incorporated a QR code to allow potential participants to scan and be directly taken to the website of an official government organization with a secure address to view the eligibility criteria and register their interest. Once they had submitted the forms on the website, the research coordinators would call them to confirm their interest, explain the study, and arrange a screening session via video chat to confirm their eligibility.

The study was designed to be conducted completely remotely, with all interaction between study team members and participants taking place via video chat sessions and with collection of biological samples by a licensed courier service.

### Online Screening Session

The screening session involved 3 parties (the potential participant, the study coordinator, and a witness who may be another member of the study team or a participant’s spouse, relative, or friend) engaged in a video call when the participant was at approximately 27 (range 24-30) weeks’ gestation. If the woman was agreeable to taking part after a detailed explanation of the study, a remote electronic signing of the informed consent form was then undertaken by all 3 parties within the same sitting. The eligibility of the potential participant was confirmed according to the inclusion and exclusion criteria (Textbox 2) and immediately recorded on the electronic research database with each participant assigned a unique participant identification code.

**Inclusion and exclusion criteria.**

**Inclusion criteria**

- Pregnant women aged ≥21 years at recruitment
- Willing and able to provide written informed consent
- Gestation of 28 to 32 weeks at randomization
- Singleton pregnancy at recruitment
- Able to complete questionnaires in English
- Hospital Anxiety and Depression Scale [28] score of ≥5 (out of 21) for either depression or anxiety subscale to indicate some general feelings of low mood or stress; each participant to fill out the questionnaire using a secured app with the scores programmed to be computed instantly and automatically upon submission
- Intention to breastfeed

**Exclusion criteria**

- Not willing or unable to comply with the study procedures and requirements
- Diagnosed food allergy
- Has taken probiotic supplements in the 4 weeks before screening
- Has received pharmacological treatment for anxiety or depression in the 12 weeks before recruitment
- Major complications during pregnancy (eg, pre-eclampsia, gestational diabetes requiring insulin intervention, severe intrauterine growth restriction, and fetal anomalies)
- Preexisting medical conditions (eg, hypertension, diabetes mellitus, thyroid diseases, autoimmune diseases such as systemic lupus erythematosus, antiphospholipid syndrome, and other major chronic illness)
- Active participation in another clinical trial or ongoing observational study

### Randomization Procedure and Study Arms

**Figure 1:**
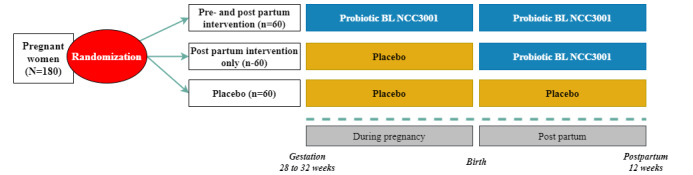
Graphical representation of the 3-parallel-arm study design. Participants are randomly allocated to 1 of the 3 different arms of the trial in a 1:1:1 ratio, with the probiotic intervention taken either (1) during pregnancy and post partum (n=60) or (2) post partum only (n=60) compared with (3) a placebo control group (n=60). BL: Bifidobacterium longum.

This is a double-blind, placebo-controlled, randomized, and 3-parallel-arm study aimed at assessing time trends in perinatal mood and stress with ingestion of the probiotic BL NCC3001, starting from either the third trimester (28-32 weeks’ gestation) or immediately after birth until 12 weeks post partum, compared with placebo. The 3 arms are (1) prepartum + postpartum probiotic, (2) prepartum placebo + postpartum probiotic, or (3) prepartum + postpartum placebo in a 1:1:1 ratio (Figure 1). Participants were randomly allocated via an electronic database to 1 of these 3 arms at the first virtual study session when the participant was at 30 (allowed window 28-32) weeks’ gestation.

Until the database lock of the primary outcome, neither the investigator nor the participant nor the site team nor the independent monitor managing the study will know to which investigational product each participant was allocated, with the exception of specified delegated regulatory staff within Nestlé, the product manufacturing site, and the supply and quality managers at the sponsor’s clinical research unit.

### Intervention

The daily intervention comprised a dry powder stick pack containing 1.0 × 10^10^ colony-forming units of the probiotic strain BL NCC3001 (8.25%) premixed with a maltodextrin (91.75%) carrier. It is being compared against a placebo of similar sensory characteristics, packaged in a dry powder stick pack containing maltodextrin (84%), yeast extract (12%), pea flour (2%), and cysteine (2%). Both products need to be stored refrigerated and were couriered to participants by designated services trained to maintain the cold chain. The participants were instructed to first dissolve the probiotic in an approved liquid medium (eg, plain water or milk) at room temperature or cooler, then consume it within an hour, in the evening with a meal on a daily basis.

The dosage and schedule were identical to those of a previous study [23], and this approach has been shown to induce an immunologic or microbiota host response to the probiotic in healthy human adults [29]. BL NCC3001 has also been administered at a similar dose to pregnant and lactating women [30,31] as well as infants [32] in studies unrelated to PMD, with no major adverse effects reported.

Adherence was assessed by participant recall at each study session. Good adherence was predefined as at least 80% over the study period. Upon the resupply or return of unused products, product consumption was additionally verified.

Usual consumption of foods containing probiotics was recorded at baseline (screening session) and again at the end of the study. Participants were requested not to increase the intake of probiotic foods across the duration of the study. In any case, the intake of particular diets, treatments, medications, or concomitant therapies such as probiotic supplements and antibiotics that may interfere with the intervention, as well as medications known to affect mood and stress, during the trial period were captured.

### Study Outcomes and Other Assessments

The primary outcome is change in time trends of mood and stress scores as measured by the STAI [24] and EPDS [25] evaluated across 6 study sessions between baseline at 28 to 32 weeks’ gestation and 12 weeks post partum.

The secondary outcomes will be derived from validated self-reported questionnaires, including probable depression requiring follow-up (defined as an EPDS score of ≥13) at each time point; change in Hospital Anxiety and Depression Scale score [28] (anxiety and depression subscales) from baseline to post partum; Parenting Stress Index [33] score at 12 weeks post partum; Pittsburgh Sleep Quality Index [34] at 36 weeks’ gestation and 12 weeks post partum; a longitudinal assessment of the Gastrointestinal Symptom Rating Scale score (explores any experience of bloating, rumbling, flatulence, and abdominal pain, as well as stool frequency and consistency) [35]; and a longitudinal assessment of the duration, initiation, and exclusivity of breastfeeding as well as breastfeeding practices using the Early Feeding Questionnaire. Other secondary outcomes will be derived from digital measures and biological sample analyses, including longitudinal changes in stress biomarkers (assessed using a digital app linked to the central electronic database and morning salivary cortisol measures) and in maternal gut microbiota (composition and probiotic strain colonization to generate mechanistic insights). The digital stress app is designed to use transdermal optical imaging of the face to measure heart rate variability and blood flow, which is then extracted and used to generate a *stress index* score on a scale ranging from 1 to 5, where higher scores reflect greater stress levels [36]. Interviewer-administered questionnaires specifically designed for this study were used to (1) capture sociodemographic and medical history, (2) record probiotic food and medication intake, and (3) record pregnancy complications and birth outcomes. Scores from a validated self-administered Childbirth Experience Questionnaire [37] were also captured because this could potentially influence postnatal mood. The timing and activities at each of the 6 study sessions are listed in Table 1.

**Table 1:** Study sessions and procedures.

Procedure				Screening session during pregnancy: gestation 27 weeks (+3 or −3 weeks)		Treatment period										
						During pregnancy				Post partum						
						Session 1: gestation 30 weeks (+2 or −2 weeks)		Session 2: gestation 36 weeks (+2 or −2 weeks)		Session 3: post partum 9 days (+5 or −5 days)		Session 4: post partum 4 weeks (+7 or −7 days)		Session 5: post partum 8 weeks (+7 or −7 days)		Session 6: post partum 12 weeks (+7 or −7 days)
Informed consent				✓												
Inclusion and exclusion criteria				✓												
HADS^a^				✓						✓						✓
Demographics				✓												
Obstetric history				✓												
Medical history				✓												
Randomization						✓										
**Biological sampling**																
		Salivary cortisol sampling			✓		✓		✓		✓		✓		✓	
		Maternal fecal sampling			✓		✓				✓				✓	
Stress index via digital app						✓		✓		✓		✓		✓		✓
**Questionnaires**																
	EPDS^b^				✓		✓		✓		✓		✓		✓	
	STAI^c^				✓		✓		✓		✓		✓		✓	
	Probiotic food intake				✓										✓	
	PSI^d^														✓	
	PSQI^e^						✓								✓	
	EFQ^f^				✓				✓		✓		✓		✓	
	CEQ^g^								✓							
	Birth events								✓							
	GSRS^h^				✓		✓		✓		✓		✓		✓	
**Investigational product**																
	Intervention dispensing and return				✓				✓						✓	
	Intervention intake				✓		✓		✓		✓		✓		✓	
	Adverse event collection				✓		✓		✓		✓		✓		✓	
	Concomitant therapies				✓		✓		✓		✓		✓		✓	
	Adherence						✓		✓		✓		✓		✓	

^a^HADS: Hospital Anxiety and Depression Scale.

^b^EPDS: Edinburgh Postnatal Depression Scale.

^c^STAI: State-Trait Anxiety Inventory.

^d^PSI: Parenting Stress Index.

^e^PSQI: Pittsburgh Sleep Quality Index.

^f^EFQ: Early Feeding Questionnaire.

^g^CEQ: Childbirth Experience Questionnaire.

^h^GSRS: Gastrointestinal Symptom Rating Scale.

All sessions were conducted virtually (e-sessions) using video or voice calls accessed via a secure link that maintains data privacy. Participants filled out self-administered questionnaires on their own devices through Medidata Patient Cloud (Medidata Solutions Inc), an electronic patient-reported outcome app that was automatically integrated with the web-based research database (iMedidata; Medidata Solutions Inc). Each study participant was provided with a deidentified personal username and password to access the electronic patient-reported outcome system. For interviewer-administered questionnaires, the study team entered the answers directly into iMedidata. Each participant was assigned a clinical research coordinator for the entire duration of her participation to help build rapport and increase participant retention; regular contact included reminders for upcoming study sessions and the accompanying activities. If a participant became noncommunicative, attempted follow-up was carried out via SMS text messaging or email, and the case was only classed as lost to follow-up or withdrawn after the window period for her final study session had passed.

### Ethics Approval, Study Conduct and Registration, and Participation

Ethics approval was granted by the A*STAR institutional research board (2020-065), and all women provided signed informed consent. This study is being conducted in line with Good Clinical Practice [38] and the Declaration of Helsinki [39]. The study has been registered on ClinicalTrials.gov (NCT04685252). The clinical data management systems developed for this study complied with Good Clinical Practice [38], predicate rule requirements, and laws and regulations (personal data protection), and they allow an audit of actions by users. Participants were reimbursed for their participation in the form of electronic vouchers that could be redeemed at a local chain of malls. Reimbursement was prorated based on the number of study sessions and tasks completed, with a maximum possible value of SGD $330 (approximately US $240) for the entire trial duration for participants who completed all activities.

### Biological Sample Collection

Saliva and stool samples were self-collected at participants’ residences and couriered back to the study site by a designated, reliable, and licensed courier trained to respect the *cold chain* to maintain sample integrity. Biological samples are being stored at –80 °C at an appropriately regulated tissue repository until the planned batch analyses at the end of the study. This strategy required comprehensive collection kits to be sent to participants, which included a clear, illustrated, and concise manual, as well as items to alleviate any concerns about needing to store the biological samples in their freezer between the time of collection and courier pickup. These items included laboratory-grade biohazard bags and additional containers to contain the biohazard bags to have multiple degrees of separation between the collected biological sample and the food items in the freezer.

### Monitoring and Safety Reporting

Monitoring was carried out periodically by an external, independent monitor. This allowed the evaluation of the trial progress, verification of the accuracy and completeness of electronic case report forms, and resolution of any inconsistencies in the trial records, as well as ensured that all protocol requirements, applicable local laws, International Council for Harmonisation of Technical Requirements for Pharmaceuticals for Human Use [38] guidelines, and investigator obligations were fulfilled.

Safety reporting was conducted in accordance with a preagreed study safety monitoring plan. All adverse and serious adverse events were recorded in the study database.

### Statistics

This is an exploratory study of the effect of BL NCC3001 on mood in pregnant and postpartum women; therefore, effect sizes are unknown. However, illustrative power calculations are shown in Table 2. Once completed, this trial will allow a potential follow-up trial to be designed and powered properly, where observations could be confirmed.

**Table 2:** Illustration of error rates and statistical power for the Edinburgh Postnatal Depression Scale (EPDS) and State-Trait Anxiety Inventory (STAI) over 1000 simulations.

Outcome and hypothesis		Comparison	Max-*t* test^a^, %
**EPDS**			
	Null	Prepartum + postpartum probiotic vs placebo control	1.70
	Null	Postpartum-only probiotic vs placebo control	1.60
	25% difference	Prepartum + postpartum probiotic vs placebo control	72.00
	25% difference	Postpartum-only probiotic vs placebo control	48.10
**STAI**			
	Null	Prepartum + postpartum probiotic vs placebo control	2.20
	Null	Postpartum-only probiotic vs placebo control	1.30
	25% difference	Prepartum + postpartum probiotic vs placebo control	69.20
	25% difference	Postpartum-only probiotic vs placebo control	50.30

^a^Rows where the hypothesis is null display the error rate, whereas the ones where the hypothesis is 25% difference in time trends display the power of the test. For the control group, the means and SDs of mood scores were based on previous perinatal studies of longitudinal Edinburgh Postnatal Depression Scale scores [40] and assumed to be normally distributed. For the prepartum + postpartum intervention group, the test scores are assumed to be gradually decreasing over time with a maximal difference of 25% with respect to the controls. For the postpartum probiotic intervention group, the effect was assumed to have the same kinetics but with onset at birth. The correlation between study sessions was assumed to be 0.5.

The trial has 2 primary outcome variables (EPDS and STAI scores) and 2 primary group comparisons (prepartum + postpartum intervention vs placebo control and postpartum-only intervention vs placebo control). Thus, the false positive rate controlled at 5% level is as follows: α / 4 = 0.0125 (for each combination of a primary outcome variable and a group comparison). The time trends for comparing prepartum + postpartum intervention versus control (session 2-session 6; Table 1) and for comparing postpartum-only intervention versus control (session 3-session 6; Table 1) are modeled by 2 separate mixed models where baseline scores (session 1; Table 1), time point (considered a categorical variable), treatment, and the interactions between the time point and treatment were fixed effects, and participant was included as a random effect (random intercept). A history of previous pregnancy complications will be added as a binary covariate to the model. The global test for time-trend differences between groups is a max-*t* test. For each group comparison, the multiplicity coming from comparison at multiple time points is dealt with by performing inference based on the joint distribution of the test statistics from the appropriate set of linear contrasts of the linear mixed model parameters (using the R library *multcomp*).

A simulation using R statistical software (version 3.6.1; R Foundation for Statistical Computing) showed a power of 50% to 70% for observing statistically different time trends when considering recruiting 60 individuals per group, after accounting for an expected dropout rate of 20%, representing 48 participants per group completing the study (Table 2).

With the substantially lower observed dropout rate of 2.7% compared with the initially anticipated 20%, more participants than expected have completed the study, and the power therefore is increased from this initial illustration. Although every effort has been made to obtain complete data sets, the mixed model used for analysis is robust against missing values and valid under missing at random. Outliers will be detected before unblinding, with appropriate action taken considering the medical context. The primary analysis will be an intention-to-treat analysis (considering all randomized participants who took at least 1 dose of the assigned product and provided postrandomization data) with a secondary per-protocol analysis planned. No interim analysis was performed for this trial.

## Results

### Recruitment and Retention

**Figure 2:**
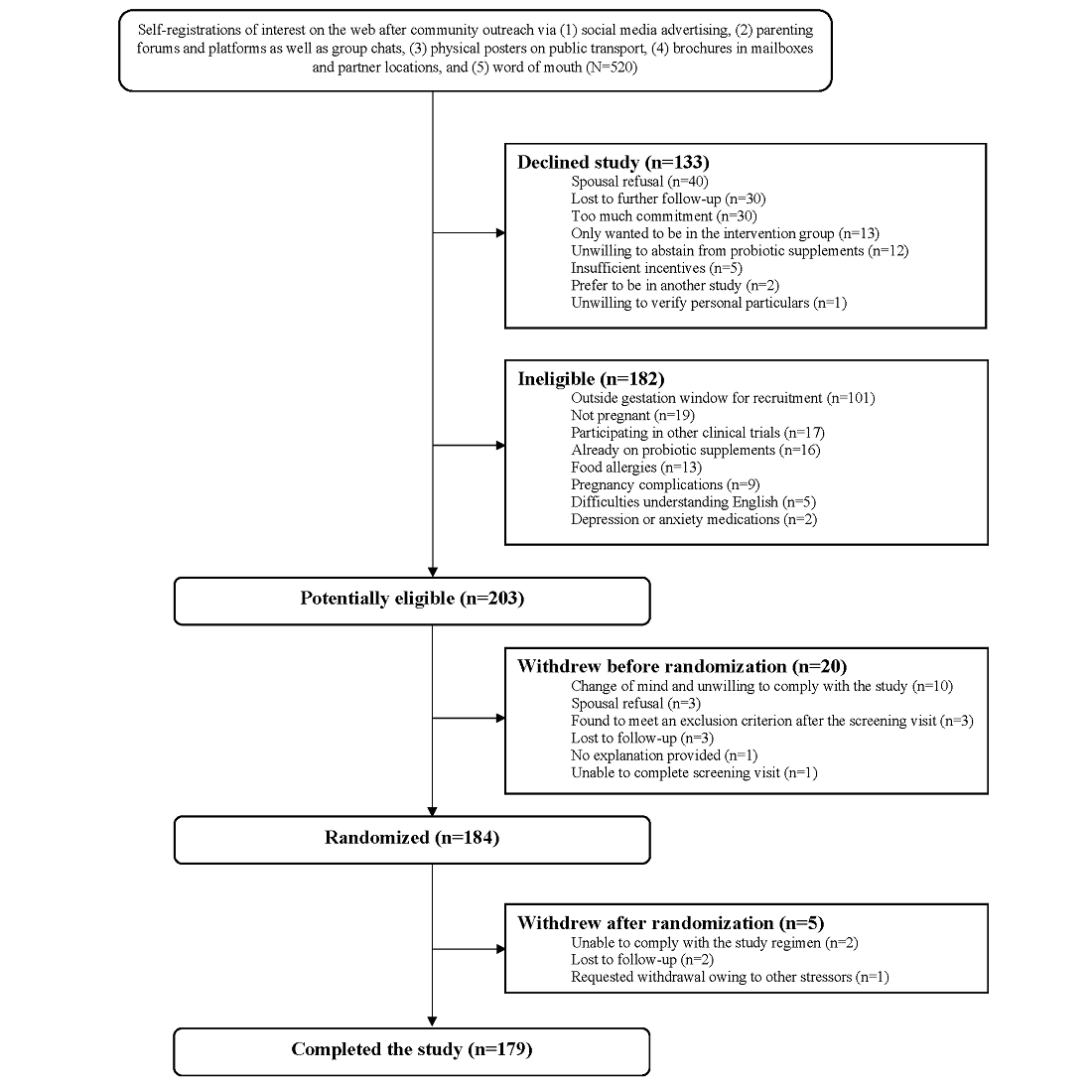
CONSORT (Consolidated Standards of Reporting Trials) diagram showing participant flow through the different phases of the trial, from expressions of interest and randomization to expected trial completion.

Recruitment for the trial commenced on November 7, 2020, and was completed in the following 10 months, with the last participant recruited on August 20, 2021. A total of 520 women expressed interest in the trial through online registration, of whom 205 (39.4%) were agreeable and potentially eligible to participate. Of these 205 participants, 21 (10.2%) withdrew or were found to be ineligible after the screening session, leaving 184 (89.8%) women who were randomized. Of these 184 women, 5 (2.7%) withdrew after randomization, and 179 (97.3%) completed the study (Figure 2). Therefore, we exceeded our target of at least 48 participants per group completing the study (the original target for the 3 groups was 48 × 3 = 144 in total) because the final attrition rate was 2.7% instead of the anticipated 20%.

### Recruitment Strategies

Of all the recruitment strategies used (Table 3), advertisements on major general social media platforms were the avenues that most women expressing interest claimed were their first knowledge of the trial (244/520, 46.9%), followed by other parenting-specific web-based platforms (116/520, 22.3%). None of them claimed to have been attracted via advertisements on the main popular search engines despite efforts to target female users of reproductive age. The recruitment avenue with the next highest success rate was via physical posters on public transport (64/520, 12.3%). The local metro train system, known as Mass Rapid Transit (MRT), is used daily by 2.2 million passengers [41], which is equivalent to approximately 41% of the population of Singapore [42].

**Figure 3:**
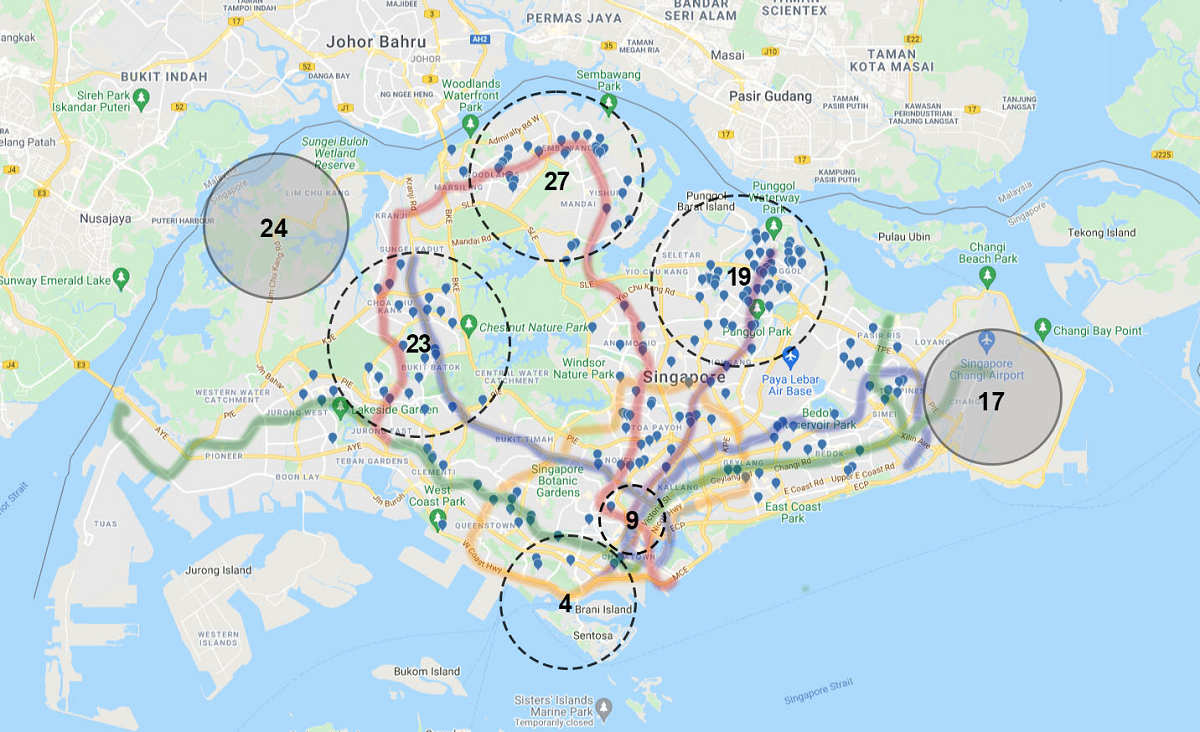
Distribution of participants’ area of residence with Mass Rapid Transit (MRT) route map of Singapore (map data: Google, 2022). Each blue drop represents 1 potentially eligible participant. Participants were recruited from across the country, particularly from districts with the highest density of residents aged <5 years (dotted circles: districts 4, 9, 19, 23, and 27) and along targeted MRT lines (purple: North-East Line, blue: Downtown Line, red: North-South Line, and green: East-West Line). Two of the 7 districts with the highest number of residents aged <5 years (districts 17 and 24) contributed no participants; in addition, they were not served by an MRT line.

The geodemographic data of Singapore were considered so that resources could be focused on the MRT routes most used by our target population. We first targeted the North-East Line, which services District 19, a catchment area that includes an area with the highest number of residents aged <5 years, an indicator of young families. Almost a quarter (122/520, 23.5%) of the prospective participants reported residing in District 19 [43,44]. The strategy was then extended to other major lines, including the Downtown Line, North-South Line, and East-West Line, that serve other districts with young families [43]. Of the 28 Singapore districts, 7 (25%) are known to have the highest proportions of residents aged <5 years; of these 7 districts, 5 (71%) contributed nearly half (233/520, 44.9%) of the potentially eligible participants, whereas the other 2 (29%; Districts 17 and 24) did not contribute any participants and were incidentally not covered by an MRT line (Figure 3).

**Table 3:** Source of recruitment advertisement reported by potential participants during self-registrations of interest on the web (N=520).

Source of recruitment advertisement or study recruitment strategy	Potential participants, n (%)
Social media	244 (46.9)
Parenting-specific online platforms	116 (22.3)
Public transport (Mass Rapid Transit trains)	64 (12.3)
Word of mouth	42 (8.1)
Physical poster or brochure	26 (5)
Internal A*STAR^a^ electronic outreach and email blast	28 (5.4)
Advertisements on search engines	0 (0)

^a^A*STAR: Agency for Science, Technology and Research.

## Discussion

### Overview

This double-blind, placebo-controlled, and randomized controlled trial will be the first to examine the efficacy of the naturally occurring probiotic BL NCC3001 in alleviating or suppressing symptoms of PMD when taken either from the third trimester or immediately after birth until 12 weeks post partum. It was designed to be conducted entirely remotely in Singapore and was successfully carried out during the height of the COVID-19–related restrictions, using digital technology in all aspects of the conduct of the study while complying fully with research governance and regulations.

Although other probiotic interventions have been tested in pregnant women for their impact on PMD with inconsistent results [16-19], it is important to note that these studies differed in one or more methodological aspects from our study. Aside from different probiotic strains or multiple probiotic strain combinations used, there were variations in probiotic concentrations; combinations with other nutritional supplements; study populations with specific comorbidities in addition to being pregnant or post partum; and different timelines for intervention intake, such as commencement in the first half of the pregnancy [17-19].

The COVID-19 era has posed many challenges globally. The first challenge concerned the need for social distancing and the associated unpredictable restrictions on in-person activities, including participation in conventional clinical trials. The second challenge concerned the fact that pregnant women are considered a vulnerable group with compromised immunity—in the early stages of the pandemic, very little was known about the detrimental effects of the virus on pregnant women and their unborn child; hence, more precautionary steps were taken to promote their isolation. Therefore, designing a research study that could overcome these hurdles and decrease barriers to participation was crucial to ensure feasibility, continuity, and the eventual successful completion of the study in the face of unpredictable governmental regulations. This study was designed to maintain robust operating procedures that would be resilient to the constant changes in COVID-19–related mandates in Singapore. This was possible because of several reasons, many novel to the local research arena. The remote approach contributed to minimizing inconvenience, reducing exposure risk to COVID-19 infection, and complying with local social distancing regulations. This included all study sessions being conducted virtually (e-visits), which comprised using digital apps to answer self-reported questionnaires and web-based databases that securely synced with these apps, and the intervention products being delivered by courier services, with self-collection of biological samples by participants conducted at their own residences and trained couriers engaged to transport the samples back, with a contactless pickup provided as an option for the participants as well. Despite the apparent success in the recruitment phase, there were some initial challenges before the study could begin. In particular, the ethics board had to be convinced of the reliability and feasibility of remote consenting procedures and approve of the entirely remote conduct of the trial, especially for a population considered vulnerable, such as pregnant women. We also had to design a new secure, acceptable, and legally valid remote e-consenting process to allow consecutive instantaneous signing by a participant, researcher, and witness located at different places, which could be completed rapidly.

The widespread ownership and use of personal mobile phone and computerized devices, high availability of internet (both cellular and home internet), trust in digital security systems associated with established local institutions, and greater IT savviness among the target age group [27], coupled with high health and research literacy, likely increased participant comfort with engaging digitally for research purposes. The established consumer trend of women of reproductive age using social media as a main avenue to research new brands, obtain information, and establish connections is another aspect that may have made the use of online channels for recruitment and conducting research in the study plausible and successful. The study team was able to achieve a much wider community reach, passively capturing a more diverse pool of participants attending different institutions for maternity care and including those mostly confined to their own home owing to COVID-19–related restrictions. The strategy of using social media proved the most effective recruitment tool because it resulted in the highest traffic of potential participants, especially during the recruitment phase that occurred from late 2020 to mid-2021 at the height of the COVID-19 pandemic when restrictions were at their most stringent in Singapore and before widespread vaccination of the pregnant population. This is in contrast to recruitment from particular health facilities (eg, a university hospital’s maternity department), which is often restrained by a geographical catchment area and the need for physical accessibility to the location to view a poster, receive a brochure, or be approached by staff to introduce the study. The same reasons that explain the success of an entirely remotely conducted clinical trial could also be used to explain the particular success of our social media recruitment strategy, despite the various other recruitment strategies being afforded similar resources in terms of time, finances, and workforce hours.

Although most of the participants (244/520, 46.9%) claimed that social media platforms gave them their first knowledge of the trial, recruitment efforts were strengthened when different avenues of recruitment were used simultaneously. Most notably, when we were targeting recruitment from a geographical perspective, interest from a particular district with a high density of young families peaked when brochures were dropped into residential letter boxes at a similar time to a physical poster advertisement campaign running on the main MRT line servicing that area. We speculate that these different forms of advertisements provided multiple reminders that increased the likelihood of expression of interest. The effectiveness of our nationwide community recruitment strategies is reflected by our data indicating that participants come from all parts of the country.

Another aspect integral to the success of the study was the at-point-of-residence self-collection of biological samples. Extensive thought and planning were required to design a biological sample collection workflow that was easy to follow, convenient, and acceptable to participants. Good coordination among the study team, participants, and the licensed courier tasked for pickup was essential. The courier had to follow a robust workflow, maintain the cold chain in tropical Singaporean weather, and demonstrate flexibility in response to constant changes in local COVID-19–related restrictions. Additional benefits that likely attracted participation include increased convenience, attributable in part to no traveling time required to attend sessions. Similarly, this also increased overall time and cost saved for the study team.

The actual subject matter of mood disorders also likely resonated more with pregnant women during the COVID-19 pandemic, which helped with study recruitment. Generally increased stress levels across the population have been widely reported worldwide during the pandemic, with pregnant women highlighted as a particularly affected group [45,46]. We aimed to recruit women who already had some symptoms of low mood or anxiety, as determined by the Hospital Anxiety and Depression Scale, to allow for the possibility of improvement with intake of the intervention product. Our data showed that none of the women who consented to join the study were excluded specifically because they did not fulfill this criterion; this means that all who were interested in participating already had some PMD symptoms. These women may have been motivated to reduce their PMD to avoid negative impacts on the health of both mother and baby and were open to exploring probiotics as a solution that is thought to have few or no unwanted effects.

As many women try to avoid taking pharmacological treatments during pregnancy and lactation [4,13], there is a potential opportunity for natural and nutrition-based solutions to provide mood benefits in this population [47]. However, a systematic review of nutritional interventions concluded that current evidence is inconsistent and inconclusive on whether nutritional factors influence the risk of PMD [48,49]. As there is some evidence suggesting that probiotics could help fill this gap [50], this study of the probiotic BL NCC3001 was designed to help inform that possibility. As the intervention had been classed by the Health Sciences Authority of Singapore as a nutrition product and not a pharmaceutical drug, it meant that the study design did not need to include the usually extensive in-person monitoring that drug trials would normally require. This, therefore, decreased the barriers to participation and study retention.

### Conclusions

Multiple converging factors contributed to the study being able to recruit at speed and retain the required number of participants despite COVID-19–related restrictions. In addition to providing novel evidence for the potential benefit of the probiotic BL NCC3001 in reducing the symptoms of PMD, this entirely remote research study design sets a precedent for similar studies. Such studies could be highly suitable if (1) the intended study population has a high rate of digital device penetration and internet access, (2) systems allow secure data exchange and capture, (3) the intervention can be self-administered without clinical monitoring, and (4) eligibility criteria and outcome measures can be reliably assessed remotely either by digital capture or self-collected biological samples. We have described here such a study that has been successfully conducted in a group considered vulnerable, pregnant women, despite the presence of challenges and barriers presented by the COVID-19 pandemic.

## Abbreviations

A*STAR: Agency for Science, Technology and Research
BL: Bifidobacterium longum
EPDS: Edinburgh Postnatal Depression Scale
MRT: Mass Rapid Transit
PMD: perinatal mood disturbances
PROMOTE: Probiotics on Mothers’ Mood and Stress
STAI: State-Trait Anxiety Inventory
